# Isoconazole and Clemizole Hydrochloride Partially Reverse the Xeroderma Pigmentosum C Phenotype

**DOI:** 10.3390/ijms22158156

**Published:** 2021-07-29

**Authors:** Farah Kobaisi, Eric Sulpice, Caroline Barette, Nour Fayyad, Marie-Odile Fauvarque, Bassam Badran, Mohammad Fayyad-Kazan, Hussein Fayyad-Kazan, Xavier Gidrol, Walid Rachidi

**Affiliations:** 1 Biomics, IRIG-BGE U1038, INSERM, Univ. Grenoble Alpes, 38000 Grenoble, France; farahkobaisi@gmail.com (F.K.); eric.sulpice@cea.fr (E.S.); xavier.gidrol@cea.fr (X.G.); 2 Laboratory of Cancer Biology and Molecular Immunology, Faculty of Sciences I, Lebanese University, Hadath, Lebanon; bassam.badran@ul.edu.lb (B.B.); mfayyadk@gmail.com (M.F.-K.); hfayyadk@gmail.com (H.F.-K.); 3 CEA/IRIG/Gen & Chem, Univ. Grenoble Alpes, 38000 Grenoble, France; caroline.barette@cea.fr (C.B.); marie-odile.fauvarque@cea.fr (M.-O.F.); 4 SYMMES/CIBEST UMR 5819 UGA-CNRS-CEA, IRIG/CEA-Grenoble, Univ. Grenoble Alpes, 38000 Grenoble, France; nour.fayyad.94@hotmail.com

**Keywords:** XPC, DNA repair, UV, skin cancer, chemical screen, DNA damage

## Abstract

Xeroderma Pigmentosum protein C (XPC) is involved in recognition and repair of bulky DNA damage such as lesions induced by Ultra Violet (UV) radiation. *XPC*-mutated cells are, therefore, photosensitive and accumulate UVB-induced pyrimidine dimers leading to increased cancer incidence. Here, we performed a high-throughput screen to identify chemicals capable of normalizing the XP-C phenotype (hyper-photosensitivity and accumulation of photoproducts). Fibroblasts from XP-C patients were treated with a library of approved chemical drugs. Out of 1280 tested chemicals, 16 showed ≥25% photo-resistance with RZscore above 2.6 and two drugs were able to favor repair of 6-4 pyrimidine pyrimidone photoproducts (6-4PP). Among these two compounds, Isoconazole could partially inhibit apoptosis of the irradiated cells especially when cells were post-treated directly after UV irradiation while Clemizole Hydrochloride-mediated increase in viability was dependent on both pre and post treatment. No synergistic effect was recorded following combined drug treatment and the compounds exerted no effect on the proliferative capacity of the cells post UV exposure. Amelioration of XP-C phenotype is a pave way towards understanding the accelerated skin cancer initiation in XP-C patients. Further examination is required to decipher the molecular mechanisms targeted by these two chemicals.

## 1. Introduction

The skin is a barrier that shields our body against pathogens, mechanical, chemical and physical stress and water loss [1]. However, direct contact with the environment makes it highly susceptible to different stimulants such as xenobiotics and UV radiation that can disrupt skin cells’ metabolism [2]. Solar UV radiation is sub-classified into UVA (320–400 nm), UVB (280–320 nm) and UVC (200–280 nm). UVB irradiation, comprising 5% of solar UV radiation, generates direct damages to the DNA in the form of dimeric pyrimidine photoproducts: Cyclobutane pyrimidine dimers (CPD), 6-4 pyrimidine-pyrimidone photoproducts (6-4PP), and Dewar isomers [3]. UVB also contributes to the generation of double-strand breaks [4] via collapsing the replication forks at dimer sites [5] and triggering the formation of reactive oxygen species (ROS) [6]. The majority of UVB-induced mutations are C → T or CC → TT transitions [7]. In wild-type situation/healthy individuals, these lesions can be removed by a process termed nucleotide excision repair (NER). The latter is divided into global genome repair (GGR) occurring throughout the genome and transcription-coupled repair (TCR) in actively transcribed genes [8]. GGR and TCR differ in their recognition step. On the one hand, XPC-Rad23B-Centrin2 and XPE -DDB1 are required for damage recognition in GGR. 6-4PP can be readily recognized by XPC while CPDs require also the cooperation of XPE-DDB1 [9]. On the other hand, CSA and CSB proteins are required for the recognition step of TCR. The following steps converge between the two sub-pathways where helicase XPD and XPB, as part of the TFIIH, are recruited to allow unwinding of the region around the damaged bases. XPA is then employed for damage verification while the nucleases XPF and XPG mediate the incision 5′ and 3′ to the damage allowing its removal, thus creating a gap. This gap is then filled by synthesizing damage-free DNA via polymerases and is eventually ligated, thus sealing the nick [10]. Failure of this repair mechanism leads to the accumulation of DNA damage, generation of mutations and cell transformation. Hereditary mutations in one of the NER proteins can hinder the repair process and lead to the generation of a wide range of diseases. Mutations in TCR sub-pathway underlie the origin of Cockayne Syndrome (CS) and UV-Sensitive Syndrome (UVSS), while patients with *Xeroderma Pigmentosum* (XP), trichothiodystrophy (TTD), XP-CS, XP-TTD and Cerebro-oculo-facio-skeletal Syndrome (COFS) are lacking proficiency in either GGR or both sub-pathways. Some of these diseases are characterized by an early onset of skin tumors. Several studies indicated that the origin of skin tumorigenesis could be mutations in different genes: *TP53* for squamous cell carcinoma [11], hedgehog signaling genes for basal cell carcinoma [12] and BRAF and RAS genes for melanoma [13]. It is thus an expected outcome for NER deficiency to be associated with skin carcinogenesis due to the mutagenic profile of the accumulated DNA damage and the UVB characteristic mutational profile seen in genes involved in carcinogenesis.

*Xeroderma Pigmentosium* C (XP-C) is a rare autosomal recessive genodermatosis. XP-C patients carry a mutation in the NER DNA damage recognition protein recognizing helix distortions opposite to pyrimidine dimers, notably 6-4PP distorting the helix at a higher angle compared to CPDs [14]. This mutation generates a diseased phenotype characterized by extreme photosensitivity and the accumulation of UV–DNA lesions. The onset of skin cancer is early in XP-C patients, often in childhood, who present a 2000 and 10,000 fold increase compared to healthy individuals in the risk of melanoma and non-melanoma skin cancers, respectively. Currently, there is no treatment for XP-C syndrome but only preventive measures including UV shielding and protection. In addition to its role in NER, XPC is also involved in several other DNA repair pathways or cellular mechanisms. For instance, XPC also participates in the initial phase of Base Excision Repair (BER), especially in the removal of oxidative DNA damage as in the case of XP-C deficient cells showing great sensitivity to the latter [15,16]. XPC has also a role in the regulation of cellular homeostasis. Silencing of XPC leads to a decrease in catalase activity leading to an increase in the levels of reactive oxygen species [17]. In addition, another study demonstrated that the accumulation of damage due to XPC deficiency increases the activation of DNA-dependent protein kinases ultimately leading to the activation of AKT1 [18]. The latter leads to the activation of NADPH oxidase1 (NOX1) which produces ROS [19]. XPC was also reported to be involved in transcription regulation. Bidon et al. demonstrated that XPC, even in the absence of DNA damage, interacts with E2F1 favoring the recruitment of ATAC (histone acetyl transferase) complex to gene promoters, thus conveying to XPC the title of RNA polymerase II cofactor [20]. These additional functions of XPC can explain the fact that XP-C patients also exhibit tumors in non-photo-exposed areas where the tumorigenesis is not linked directly to UVB induced DNA damage pointing out towards other tumorigenic pathways mediated via XPC [21].

In this study, we screened a library of approved FDA and EMA chemical drugs on XP-C patient-derived fibroblasts with the aim to identify compounds that could help in normalizing or at least ameliorating the XP-C-associated cell phenotype following UV exposure. We identified two drugs, isoconazole and clemizole hydrochloride, that can partially reverse this phenotype. This attempt of drug repurposing aims at the identification of new roles for existing drugs whose pharmacodynamics, pharmacokinetics and toxicology characteristics are already well established, thus speeding up the benefits of the use of these drugs for a new therapeutic purpose for XP-C patients who do not have any treatment option presently.

## 2. Results

### 2.1. Characterization of XP-C and Wild-Type (WT) Fibroblasts Used in This Study

#### 2.1.1. XPC Protein Expression Is Lost in XP-C Cells Compared to WT Cells

**Figure 1:**
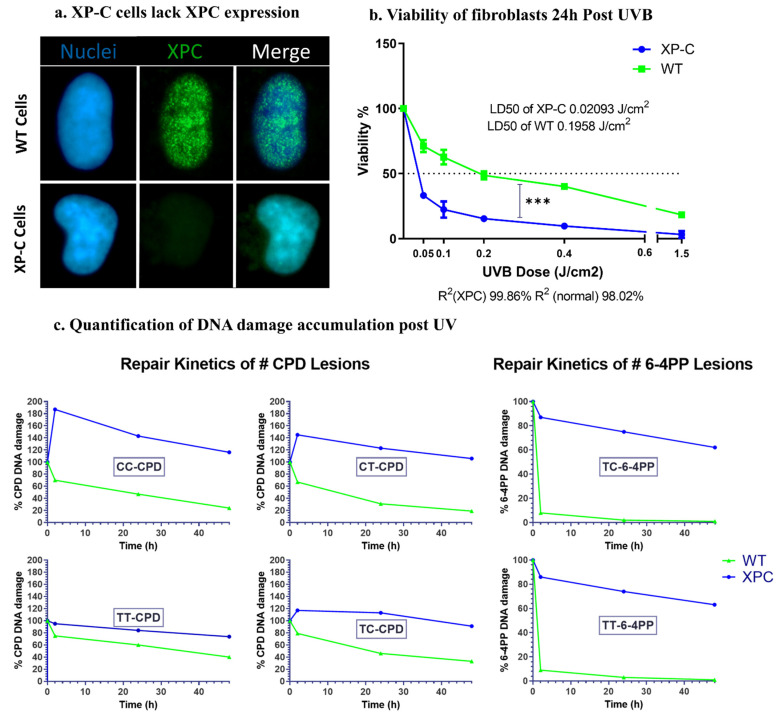
Characterization of XP-C and WT cells. (**a**) XP-C cells lack the expression of XPC protein. XP-C and WT cells were fixed and stained with anti-XPC antibody to analyze its differential expression in both cell lines. XP-C cells, unlike WT cells, do not manifest XPC protein expression in their nuclei. (**b**) Viability of fibroblasts 24 h post UVB. XP-C cells manifest significantly increased photosensitivity compared to WT cells. Both XP-C and WT cells were seeded in 96-well plates to be irradiated at 80% confluency with increasing UVB doses, then their viability was quantified 24 h later by the incubation with PrestoBlue. XP-C cells show a sharper significant decrease in viability as a function of increased UVB dose compared to WT cells. Viability was calculated by means of percent of control with 100% control being non-irradiated cells. *** *p* < 0.001, unpaired *t*-test. Results presented are the mean of three technical replicates ± SEM. (**c**) Quantification of DNA damage accumulation post UV. WT cells manifest faster repair kinetics of both CPD and 6-4PP lesions post UV compared to impaired repair in XP-C cells. Both cell lines were subjected to UVB then incubated for different time points post UV. At each time points, cells were collected, their DNA extracted and digested to be then analyzed by LC-MS/MS. Four different CPD lesion types were quantified with TT-CPD being the most frequent. Normal cells show a decrease in the % of CPD lesions as a function of time signifying repair with different kinetics per lesion time. XP-C cells, however, manifest an increase in CPD lesion at 2 h post UV and continue to have a large amount of lesion % as time elapses, a sign of impaired repair. Two lesion types were quantified for 6-4PP with TC-6-4PP being more abundant. Similar to CPD, impaired repair is visualized in XP-C cells manifested by a slow decrease in lesion % as a function of time compared to the normal cells, which in contrast to CPD lesions show even faster repair for 6-4PP. CPD: cyclobutane pyrimidine dimer, 6-4PP: 6-4 photoproducts, WT: wild type, XP-C: Xeroderma Pigmentosum C, LD50: lethal dose 50, R^2^: coefficient of determination.

The expression of XPC protein was examined in both WT and XP-C cells immortalized from patient primary fibroblasts. Immuno-staining of both cells using an antibody against the XPC protein was carried out. In contrast to WT cells, XP-C cells showed a total absence of XPC protein (Figure 1a).

#### 2.1.2. Increased Photosensitivity of XP-C Cells in Response to UVB Irradiation Compared to WT Cells

To examine the photosensitivity of XP-C cells relative to WT, both kinds of cell lines were seeded until 80% confluency then subjected to increasing doses of UVB. Twenty-four hours after UV treatment, cell viability was recorded as a measurement of the cells’ reducing capacity (Presto blue, Thermofisher Scientific, Waltham, MA, USA). Viability was normalized based on the calculation of the percentage of viability compared to the viability of control non-irradiated cells set as 100% viability. Both cell lines showed a decrease in viability as a function of increased UVB doses. XP-C cells were more photosensitive compared to the WT ones showing a sharper significant decrease in viability at each UVB dose (*p* < 0.001). The UVB dose leading to 50% of mortality was determined for both XP-C and wild-type cells (LD50). XP-C cells showed a much lower LD50 (about 0.02 J/cm^2^) compared to WT cells (about 0.19 J/cm^2^) (Figure 1b). These results confirm the strong sensitivity to UVB of XP-C cells used in this study as described in the other model [22].

#### 2.1.3. Absence of XPC Impairs DNA Repair of UVB-Induced Lesions

XPC protein is essential for the lesion recognition step of GGR [23]. For that, we aimed to determine the effect of *XPC* mutation on the repair of lesions. The two major photoproducts generated after UVB exposure are CPDs and 6-4PPs that are formed between pyrimidine dimers, either TT, TC, CT or CC. The cells were seeded until 80% confluency then irradiated at 0.02 J/cm^2^, corresponding to the previously determined XP-C LD50, then collected post UVB treatment at different time points. In order to monitor DNA lesions, DNA was extracted and digested to be analyzed by LC-MS/MS. For CPDs, the four different lesion types were quantified. WT cells showed a decrease in different CPDs lesions’ amounts as a function of time to reach a minimum after 48 h indicating efficient repair of DNA damage, while in contrast, XP-C cells showed constant elevated amounts of lesions as a function of time (Figure 1c). It should be noted that not all lesions were present in the DNA in the same quantities: the majority of CPD lesions were of TT-CPD nature followed by TC-CPD, CT-CPD, while the least abundant lesion was CC-CPD in both kinds of cells. In addition, the repair kinetics in wild-type cells differed between lesion types with the fastest repair observed for the CT-CPD. Only the TT and TC lesions could be detected for the 6-4PPs, as the two remaining lesions were less frequent. 6-4PPs were repaired faster than CPD with total repair 24 h post UV in WT cells compared to the higher amount of lesions in XP-C cells. There were no discrepancies in the repair kinetics between the TT and TC 6-4PP lesions in normal cells (Figure 1c). Taken together, our results show that wild-type but not XP-C cells efficiently repair photoproducts resulting from UVB irradiation.

### 2.2. Primary Screen Identified 16 Candidate Compounds That Increase XP-C Cells Viability Post UVB Irradiation

**Figure 2:**
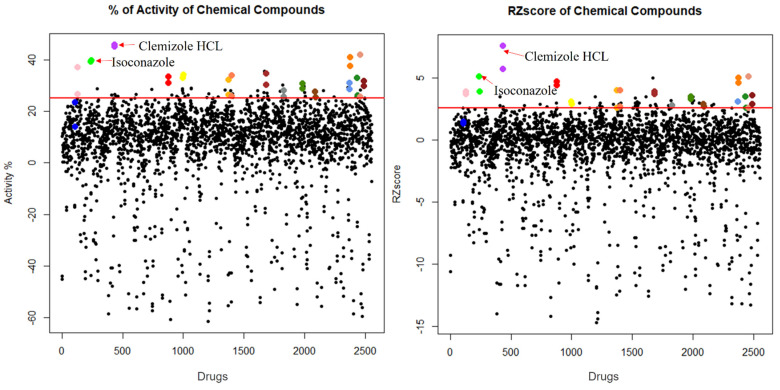
Identification of 16 compounds reversing XP-C photo-sensitivity upon UVB irradiation. Prestwick library of chemical drugs was screened on XP-C cells. The cells were treated with the library for 24 h, then further on, irradiated. Post Irradiation, the cells were then incubated with the library for an additional 24 h prior to having their viability assessed 24 h post UV via the addition of Presto Blue reagent and the recording of the fluorescent values. Based on two different normalization criteria, control based % of activity and non-control based RZscore, 16 compounds were selected manifesting >25% increase or >2.5 value for both % of activity and RZscore respectively. % Activity was calculated by normalizing the obtained values between the interval of 100% donated to non-irradiated DMSO treated cells and 0% that donated irradiated DMSO treated cells. RZscore was measured by normalizing the fluorescence values to the median and median absolute deviation. RZscore: Robust Z score.

To identify compounds that would correct the photosensitive phenotype of XP-C cells, we did set up a robotic assay in 96-well microplate to monitor cell viability after UVB irradiation (see methods and Supplementary Figure S1, see Supplementary Materials). The robustness of the assay was calculated as a Z’ factor [24] at various UVB doses on a Z’ plate including 48 bioactive controls mimicking the desired effect (here, non-irradiated protected XPC cells in DMSO) and 48 bioinactive controls (here, irradiated cells in DMSO). A library of 1280 FDA and EMA approved drugs (Prestwick Library) was then screened on XP-C cells at 10 µM for 24 h in duplicates using this assay. Briefly, following drug treatment, the cells were UVB irradiated at 0.05 J/cm^2^ based on Z’ Factor calculation (Supplementary Figure S1), then post-treated with the same drugs and incubated at 37 °C for an additional 24 h period before the measurement of cell viability. In this setup, the effect of drug treatment on cell viability can be either preventive or curative to the UVB-induced damage. The bioactivity of each tested compound, signifying the drugs’ beneficial effects on photo-resistance, was calculated by determining the fluorescence of each well with respect to the fluorescence of non-irradiated DMSO treated wells set as 100% (bioactive control) and irradiated DMSO treated wells set as 0% (bioinactive control). The robust Z score was also calculated per plate as an additional normalization technique [24]. The hit selection was based on two criteria: compounds that possess a bioactivity ≥25% and a robust Z score ≥2.5. Sixteen molecules were identified as primary hits on this basis and selected for further characterization of their bioactivity on XP cells (Figure 2).

### 2.3. Selection of Three Bioactive Drugs Increasing XP-C Cells Viability after UVB Irradiation

**Figure 3:**
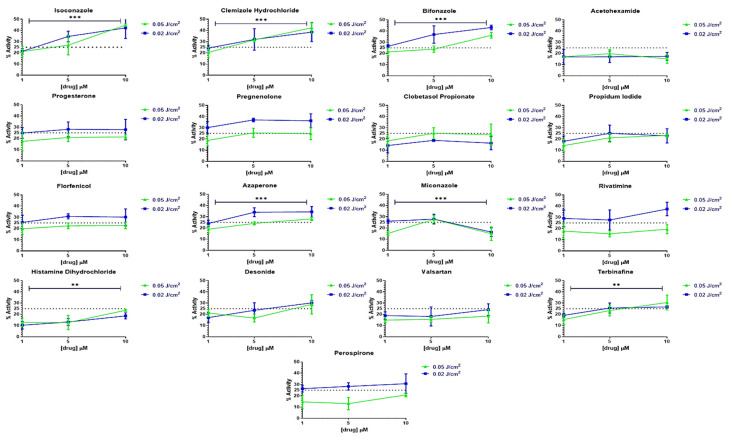
Confirmation of primary screen hits and the identification of the 3 most effective compounds. XP-C cells were treated with the different primary screen candidate compounds at three different concentrations (1, 5 & 10 µM), then subjected to two UVB doses (0.02 & 0.05 J/cm^2^) after which their viability was measured 24 h later by the addition of PrestoBlue. It should be noted that pre- and post-irradiation treatment with the drugs was carried out. The three compounds showing the increased drug bioactivity as a function of increased concentrations are isoconazole, clemizole hydrochloride and bifonazole, showing the highest protection against UV irradiation. % Activity was calculated by determining the relative % of fluorescence with respect to non-irradiated DMSO treated cells set as 100% and irradiated DMSO treated cells set as 0%. The dotted line represents a threshold of 25% Activity. The statistical significance of the effect of two UVB doses and the three drug concentration on the % activity was conducted by using 2-way ANOVA ** *p* < 0.01, *** *p* < 0.001. The figures represent triplicates ±SD.

In order to select the most efficient compounds showing robust activity on XPC viability among the 16 primary hits, a secondary screen was carried out using the same assay as the initial primary screen. Additional conditions were included together with the initial screen’s conditions. The drugs were indeed screened at three different concentrations (1, 5 and 10 µM). Moreover, the cells were irradiated at two doses of either 0.02 J/cm^2^ or 0.05 J/cm^2^. Finally, each drug was screened in triplicate in each of the different irradiation conditions and drug concentrations. The same viability readout was measured as for the primary screen. The acetohexamide drug was also added as a positive control as it was reported to increase XP-A cells’ viability in response to UV irradiation [25] where XPA is a protein involved in the damage verification process downstream damage recognition post irradiation [26]. For each drug, a dose-response curve of bioactivity as a function of drug concentration (in µM) was plotted for the different doses tested. The drugs that showed the highest bioactivity were isoconazole, clemizole hydrochloride and bifonazole, inducing a significant 20 to 40% increase in XP-C cell viability with *p*-value < 0.001 depending on the UVB exposition and drug concentration. In contrast, the acetohexamide only mediated a 20% increase in viability of XPC cells and displayed no increased bioactivity when applying increasing concentrations to the cells (Figure 3). This secondary screen defines three promising drugs protecting XPC cells from UVB toxicity.

### 2.4. Identification of Drugs That Promote the Repair of DNA Damage

**Figure 4:**
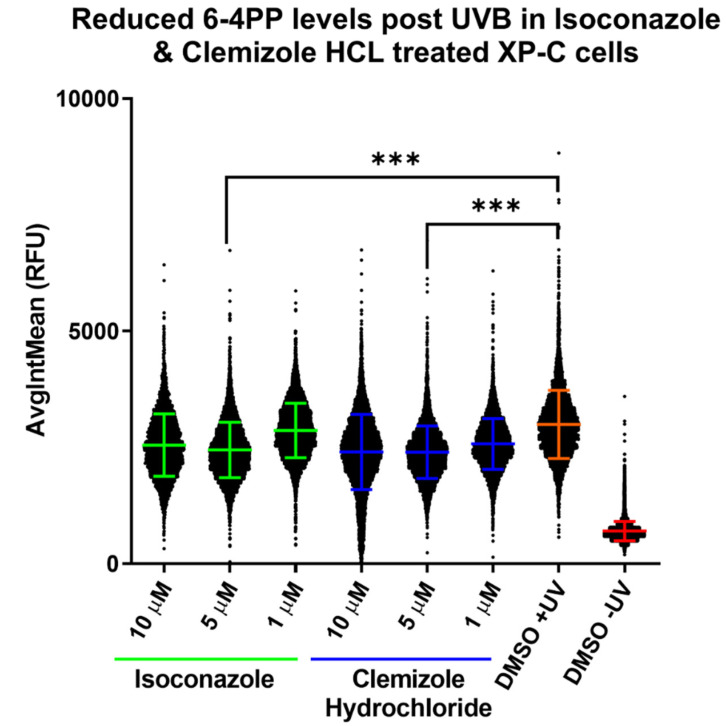
Identification of compounds enabling repair of 6-4PP in XP-C cells. XP-C cells were treated with the 16 primary screen candidate hits at three different concentrations (1, 5 & 10 µM) with pre- and post-irradiation treatments. Irradiation was carried out at a dose of 0.05 J/cm^2^. 24 h post UV the cells were fixed and stained with 6-4PP antibodies for the quantification of DNA damage. Fluorescence intensities of individual cells for each condition were quantified and more than 1000 cells per condition were analyzed. Among the 16 hits, only isoconazole and Clemizole hydrochloride enabled a decrease in the amount of DNA damage post UV shown here. Almost 20% repair of 6-4PP DNA damage was recorded for both drugs at a concentration of 5µM which was found to be significant. DNA damage quantification in DMSO treated irradiated cells was used as positive control showing the maximum amount of DNA damage while quantification of 6-4PP in DMSO treated non-irradiated cells was taken as a negative control with the minimum amount of detected damage. *** *p* < 0.001, freedman non-parametric test with Dunn’s post hoc analysis. Up to 10,000 cells were analyzed per condition with ±SD.

In order to determine the mechanisms of action of the 16 primary hits and acetohexamide, we tested whether they could promote DNA repair activity in XP-C cells. For that, DNA damage analysis was carried out after cells were treated with the 17 drugs and irradiated at a dose of 0.05 J/cm^2^ using the same protocol as for the primary screen. After the measurement of bioactivity, the plates were fixed and immuno-stained with an anti-6-4PP antibody for the quantification of the amount of DNA damage following UVB irradiation in the presence of drug or not (Supplementary Figure S2). It should be noted that the amount of DNA damage induced at time zero after UVB irradiation was the same for either treated or non-treated cells. The positive control showing maximal DNA damage was the DMSO treated irradiated cells while the negative control with minimal damage is the non-irradiated DMSO treated cells. XP-C cells treated with either isoconazole or clemizole hydrochloride showed a significant decrease in the amount of 6-4PP at the single-cell level compared to untreated controls, suggesting enhanced repair activity due to drug treatment. This decrease in DNA damage was significant at a 5 µM concentration for both drugs (*p* < 0.001) while not at 1µM, which may be a too weak concentration, or at 10 µM, where the difference was not found to be significant (Figure 4). These results thus identified isoconazole and clemizole hydrochloride as two drugs promoting DNA repair in response to UVB irradiation in XP-C cells.

### 2.5. Dose-Response Analysis of Drug Bioactivity as a Function of UVB Doses in XP-C and WT Cells

**Figure 5:**
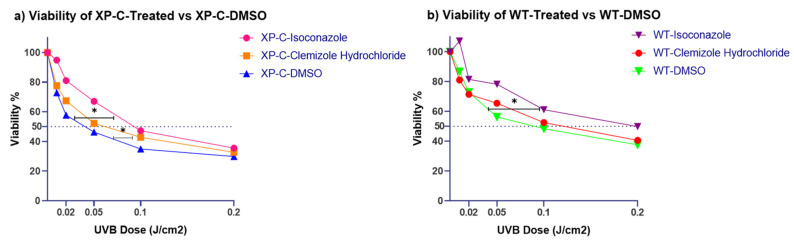
Isoconazole and Clemizole Hydrochloride viability analysis post UVB in both XP-C and WT cell lines. XP-C and WT cells were treated with either drugs or DMSO (pre- and post-irradiation treatment), then subjected to increased UVB doses. Their viability was further on assessed 24 h post UV by the incubation with PrestoBlue. (**a**) Viability of XP-C treated cells vs. DMSO. Both drugs enabled significant (*p* value < 0.05) protection of XP-C cells from UVB induced death with isoconazole having a more protective effect. (**b**) Viability of WT treated cells vs. DMSO. Drug mediated protection against UVB irradiation was also evident in treated WT cells and not unique to XP-C cells. Only isoconazole mediated protection was significant with *p* value < 0.05. Viability was calculated by determining the percent of control with respect to non-irradiated DMSO treated cells taken as 100% viable. * *p*-value < 0.05, Repeated Measure one way ANOVA with Dunnett’s multiple comparison test. The experiments were carried out in triplicates.

To further characterize the effect of isoconazole and clemizole hydrochloride drugs on XP-C cells versus wild-type cell viability enhancement, UV dose-response experiments were carried out. In contrast to the previous experiments, the dose-response analysis here was performed on both the XP-C and WT cell lines and viability was normalized to that of non-irradiated cells (fixed at 100%). This task aims to decipher whether the effect of the two drugs on cell viability is specific to XP-C cells or also valid in WT cells. The two cell lines were treated with either isoconazole, clemizole hydrochloride or DMSO, then subjected to increasing UVB doses. Both drugs enabled enhanced photo-resistance to the increasing UVB doses in both XP-C and WT compared to DMSO treated cells (Figure 5a,b). Such photo-resistance was significant for both drugs in XP-C cells with a *p*-value < 0.05. However, in WT cells, the treatment with isoconazole showed significant photo-resistance against increased UV doses irradiation (*p*-value < 0.05), unlike clemizole hydrochloride whose increase in viability was not significant. In conclusion, this protective effect may not be specific to XP-C cells as a similar protective effect was also shown for the WT cells. This is consistent with their observed ability to increase DNA repair efficiency.

### 2.6. Investigation of Pre- Versus Post-Drug Treatment Efficacy

**Figure 6:**
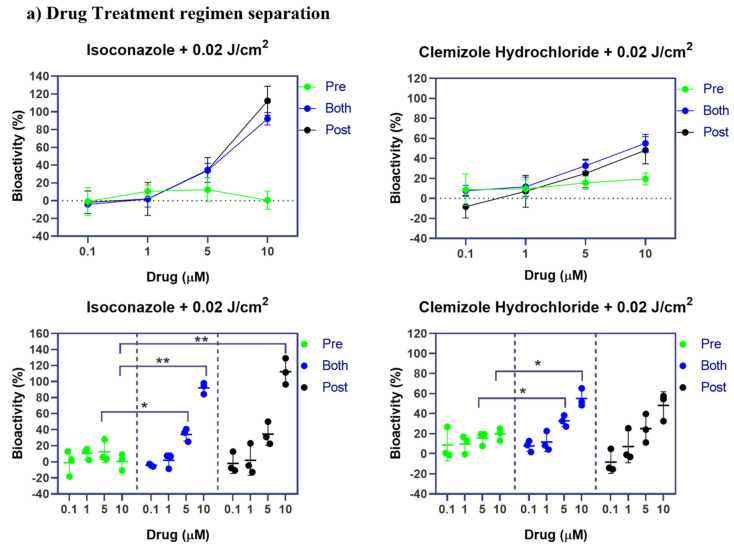

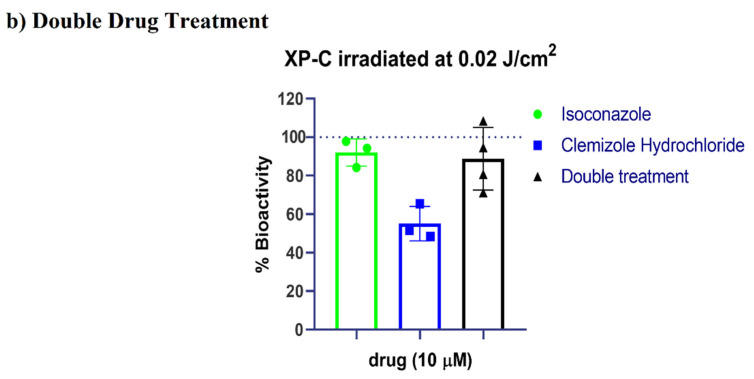
Drug treatment regimen separation and double drug treatment. To determine which treatment regimen was more effective, XP-C cells were treated with the drugs either pre-irradiation, post-irradiation or both pre and post-irradiation. Drug Bioactivity % was then calculated 24 h post UV. Such calculation is based on determining the relative percentage considering non-irradiated DMSO treated samples as 100% and irradiated ones as 0%. Thus, the obtained fluorescence values normalized to these two controls. (**a**) Drug treatment regimen separation. Isoconazole post-irradiation or both treatments showed a significant *p* value < 0.01 increase in bioactivity at 10 µM, while only both treatments showed significant enhancement of bioactivity at 5 µM compared to pre-treatment procedure. Clemizole hydrochloride, however, seems to have better bioactivity when both pre and post-irradiation regimes were used where the increase of bioactivity was significant, *p* value < 0.05 for both 5 µM and 10 µM treatment compared to pre-treatment at each concentration, respectively. Moreover, double drug treatment was also carried out to test whether both drugs possess synergistic or additive effect. (**b**) Double drug treatment. XP-C cells were treated with either Isoconazole, clemizole hydrochloride or double treated at 10 µM, then irradiated at 0.02 J/cm^2^. % of drug bioactivity was measured 24 h later. Double treatment showed the same protective profile as isoconazole with no added protection against UVB irradiation. * *p* value < 0.05, ** *p* value < 0.01, 2-way ANOVA with Tukey’s multiple comparison test. The experiments were performed in triplicate with ±SD.

As mentioned above, the chemical treatment consisted of two phases, a pre-irradiation treatment to test the preventative effect of the drug and a post-irradiation treatment to examine its curative effect. In order to determine which treatment phase had a more essential role in the XP-C cells phenotypic normalization, the two phases of treatment were analyzed separately via the determination of drug bioactivity. It should be noted that bioactivity is defined by the ability of the drugs to impose an effect on living matter, which in this case, is its ability to increase photo-resistance of the cells between non-irradiated cells set at 100% and irradiated non-treated cells set at 0%. The effect of each of the pre- or post-treatment was compared to that of the combined pre- plus post-treatment regimen. For isoconazole, the post-irradiation treatment was sufficient to induce similar protection as the combined treatment at both 5 and 10 µM (with no significant differences) while the pre-treatment had no effect on XP-C cells viability at any of the tested concentrations (Figure 6a). At 10 µM, the difference in bioactivity was significant between pre and post-treatment as well as between the pre-and combined treatment with a *p*-value < 0.01, signifying that both pre-treatment and combined regimens share significant protection compared to pretreatment. In the case of clemizole hydrochloride, the pre-irradiation treatment showed slight bioactivity reaching 20% increased cell viability at the highest concentration of drug of 10 µM. The post-treatment, however, was again more effective than the pre-treatment on XP-C viability reaching an average of 50% increase of cell viability at 10 µM. The combination of both pre-and post-irradiation treatments showed the highest bioactivity with a significant difference compared to the pretreatment at both 5 and 10 µM *p*-value < 0.05, and no significant difference with the post-treatment only. Thus, despite a slight protective potential of clemizole hydrochloride, both drugs (isoconazole and clemizole hydrochloride) may essentially serve as curative remedy post-UVB irradiation.

### 2.7. Double Drug Treatment Has no Synergistic nor Additive Effect

Both compounds, isoconazole and clemizole hydrochloride, have an azole ring in their structure (and may thus target the same molecular biological target or similar mechanisms). We, therefore, examined whether the combined treatment with both drugs at 10 µM could improve further or not the acquired photo-resistance compared to single-drug treatment. Accordingly, XP-C cells were treated with either drug alone or with both and then subjected to irradiation at a dose of 0.02 J/cm^2^. The double treatment had the same bioactivity as the single treatment with isoconazole with no benefit (Figure 6b). Hence, no synergetic effect was obtained upon double drug treatment.

### 2.8. Isoconazole and Clemizole Hydrochloride Do Not Affect Cell Proliferation

In a first step, and in order to analyze the proliferative state of the irradiated cells and whether the different treatments can modify this profile, cells were stained with Ki67 antibodies and analysis was carried out at the single-cell level. Ki67 antigen was expressed during all phases of the cell cycle (G1, S, G2 and M) but not in quiescent cells. Both XP-C and WT cell lines were positive to Ki67, signifying that the cells are not in quiescent state post UV. Cells treated with either DMSO, isoconazole, clemizole hydrochloride or both drugs showed no difference in the Ki67 expression profile (Supplementary Figure S3).

**Figure 7:**
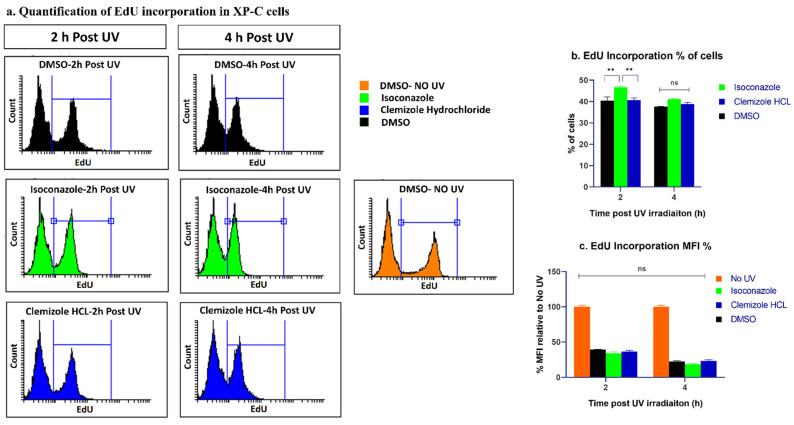
Same EdU incorporation profile is evident between drugs or DMSO treated XP-C cells. XP-C cells were treated with the different drugs or DMSO, then irradiated at 0.02 J/cm^2^. Prior to the end of the post UVB incubation, the cells were incubated in the presence of EdU. Cells were further on collected, stained and analyzed by flow cytometry. No difference is seen between the mean fluorescence intensity of the EdU positive populations between the different treatment condition at either 2 or 4 h post UV. It should be noted that as incubation time post UV increases, the mean fluorescence intensity of EdU positive population decreases. Quantification of both the mean fluorescence intensity (MFI) and EdU positive population percentage were carried out. Two-way ANOVA was used to compare between the two independent variables (time post UV and nature of the drug treatment) and the dependent variable of either EdU population percentage or MFI, ** *p* < 0.01, ns: not significant. The experiments were carried out in triplicates with ±SD. (**a**) Quantification of EdU incorporation in XP-C cells, (**b**) EdU Incorporation %of cells, (**c**) EdU Incorporation MFI %.

Ki67 antibodies stain cells in various stages of the cell cycle, and it was previously reported that some non-proliferating cells tend to test positive for Ki67 due to antigen retention [27]. Moreover, bulky adducts generated by UV tend to block the progression of replication forks, decreasing DNA replication. Therefore, in a second step, EdU incorporation assay was performed to clarify whether this positive staining was due to antigen retention or whether rather the cells were capable of recovering from the DNA replication blockade following different treatments. It should be noted that EdU insertion into DNA allows the identification of cells with active DNA synthesis that generally occurs during S phase. XP-C cells were treated with either DMSO, isoconazole or clemizole hydrochloride in a pre-and post-treatment regime and then irradiated with UV to be finally cultured in the presence of EdU at different time points (2 h or 4 h post UV). Cells were then collected and stained to be analyzed by flow cytometry. When comparing the course of EdU incorporation in DMSO treated cells over the different time points post UV, it was clear that the intensity of EdU decreases as a function of time till becoming null at 24 h post UV. This signifies that the cells were no longer able to undergo DNA replication at 24 h post UV. No increase in the EdU MFI (mean fluorescence intensity) for the drugs compared to DMSO treatment was recorded (Figure 7c). Isoconazole had a slight effect on increasing EdU positive population at 2 h post UV with *p* < 0.01 (Figure 7b) which might either be attributes to a slight increase in cell proliferation or an increase in translesion synthesis allowing more DNA synthesis upon drug treatment. Both drugs, however, failed to increase DNA replication beyond that, showing similar effects to DMSO (Figure 7b). These results indicate that cell proliferation does not seem to be the major mechanism underlying the protective effect induced by these drugs.

### 2.9. Isoconazole and Not Clemizole Hydrochloride Increases Live Cell Population in the Course of Apoptosis and Necrosis Analysis

**Figure 8:**
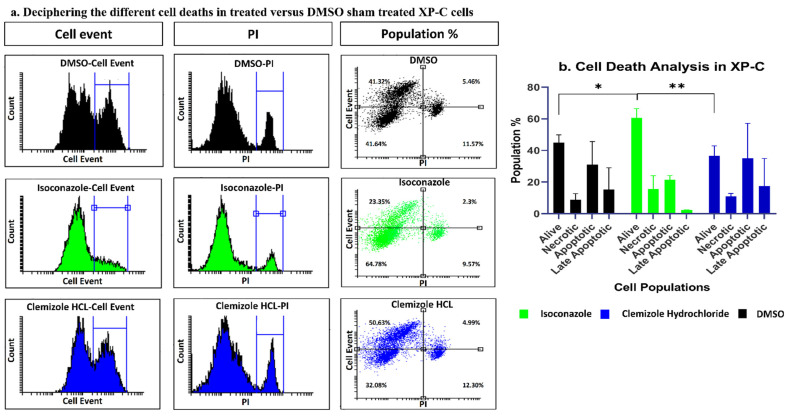
Isoconazole but not clemizole hydrochloride increases live cell population of XP-C cells post UV. XP-C cells were treated with the different drugs or DMSO then irradiated. CellEvent caspase 3/7 marker was added post UV then cells were collected. PI was added just before analysis on a flow cytometer. Isoconazole treatment decreased the percentage of apoptotic cells post UV compared to DMSO treated cells. Clemizole hydrochloride, however, had no effect so it is perhaps via another mechanism that this drug mediates its mode of action. Student *t*-test. ** *p* < 0.01, * *p* < 0.05. The experiments were carried out in triplicates with ±SD. (**a**) Deciphering the different cell deaths in treated versus DMSO sham treated XP-C cells, (**b**) Cell Death Analysis in XP-C.

In an attempt to decipher the cellular mechanisms of photo-resistance of the drug treatment, different cell phenotypes were analyzed. Apoptosis was therefore quantified using Cell Event which allows the indirect measurement of caspase 3/7 activity, key downstream players in the apoptosis activation cascade. XP-C cells were therefore treated with either drug or DMSO followed by UVB irradiation then staining with Cell Event and PI 24 h post UV. The samples were analyzed by flow cytometry. Out of the two drugs, Isoconazole showed a decrease in Cell Event % cells reaching 23.35% as well as lower PI-positive population percentage (9.57%) compared to DMSO treated cells, showing higher population percentages of 41.23% and 11.57% (Figure 8a-right panel), respectively, but was not found significant. However, the increase in the levels of the live cell population originating from this decrease in both apoptotic and necrotic populations was significantly regulated compared to that of DMSO (*p*-value < 0.05) or to that of clemizole hydrochloride (*p*-value < 0.01). On the other hand, clemizole hydrochloride did not show any decrease in both quantified parameters. These results suggest that, unlike isoconazole, it is probably not via the reduction of apoptosis that the clemizole hydrochloride mediates its protective role (Figure 8b). Similar outputs were also detected in WT cells (Supplementary Figure S4).

## 3. Discussion

The presented work identified two drugs, isoconazole and clemizole hydrochloride, that could partially reverse the XP-C characteristic phenotype: increased photosensitivity and absence of DNA damage repair of photoproducts. In addition, both drugs also enabled photo-protection in WT cells exposed to high doses of UVB irradiation.

XP-C patient cells carry a mutation in the *XPC* gene required for the recognition of DNA damage. This mutation renders the cells photosensitive [28] and unable to repair UVB induced DNA damage. Here we confirmed the photosensitive profile of XP-C as they show a significantly (*p*-value < 0.001) reduced viability compared to WT cells as a function of increased UV doses. Such cells lack the XPC proteins, as confirmed by immunostaining, signifying that such a mutation leads to the complete loss of the XPC protein. Moreover, upon the quantification of the different UVB induced DNA lesions by LC-MS/MS, it was evident that XP-C cells manifest slower repair kinetics compared to WT cells. UVB induced lesions include either CPD or 6-4PP that are formed between two adjacent pyrimidines giving four possible lesions T-T, C-C, C-T and T-C [29]. The most abundantly formed lesions are the T-T and T-C lesions for both the CPD and the 6-4PP. In WT cells, both lesion types of 6-4PP are readily repaired by the cells a few hours post irradiation, while CPDs require more time, with lesion dependent repair kinetics being the slowest repair for T-T, T-C, C-C then C-T, CPD showing the fastest repair, confirmed by us and others [30]. XP-C cells show no or reduced repair for both CPD and 6-4PP different lesion types as a function of time.

Drug repurposing is the process of testing previously approved drugs, used for a particular therapeutic purpose, for their potential use in the treatment of other common or rare diseases. This cuts down drug discovery costs and the long process of toxicity and pharmacokinetics testing [31]. For that, our aim was to test a library of 1280 approved drugs for their potential use in normalizing the XP-C phenotype. XP-C cells were treated with the compounds at 10 µM for 24 h then irradiated with UVB and then treated again with the same compounds at the same concentration for an additional period of 24 h. The overall viability of the treated cells, proportional to the amount of metabolically active cells, was then measured via incubation with PrestoBlue. The fluorescent values obtained were normalized via either the calculation of drug activity %, control based with 100% for non-irradiated DMSO treated cells and 0% for irradiated ones or via the calculation of the non-control based RZscore. Drugs that showed an activity ≥25% with an RZscore above 2.6 were selected as primary hits to be confirmed via a secondary screening which were 16 drugs. Acitohexamide was added to the secondary screen drug list due to its previously described photo-protective effect on XPA cells via the enabling of damage repair [25]. Isoconazole, clemizole hydrochloride and bifonazole manifested the highest bioactivity after secondary screening enabling photo-protection in XP-C cells compared to DMSO treated controls with a range of 20 to 40% increase of cell viability depending on the conditions. However, the enabling of photo-resistance is not sufficient by itself for a satisfying curative outcome. The persistence of cells with accumulated DNA damage can lead to the conversion of such lesions into mutations which, depending on their localizations, can lead to carcinogenesis [32]. The main lesion in XP cells mediating the UV signature mutation is the C-C lesion where the slow repair of such lesion in XP cells compared to its fast repair in WT cells favors the deamination and induction of mutations [29]. Therefore, photo-resistance should be associated with increased DNA damage repair in the perspective of therapeutic treatment. Indeed, we tested the capacity of the 16 primary hits and acetohexamide in mediating the decrease of DNA damage post UVB irradiation. Out of this collection, two compounds, isoconazole and clemizole hydrochloride, decreased the amount of DNA damage post UV in XP-C cells by about 20% (*p*-value < 0.001 at 5 µM).

Isoconazole is an azole antifungal drug with no reported data on its effect in sun-shielding upon its use on the skin, while clemizole hydrochloride is a Histamine H1 antagonist. Other documented functions for clemizole hydrochloride include its role in the treatment of HCV infection [33] and its blocking effect on TRPC5 ion channels [34]. These functions fail to explain these drugs’ effect on XP-C cells, suggesting possible alternative mode(s) of action than the ones documented that need(s) to be further on explored.

One similar approach to ours was conducted on XP-A cells, where the anti-diabetic drug acetohexamide was found to be effective in reducing the cells’ photosensitivity and enabling damage repair [25], mediating the identification of an additional mode of action for this drug not related to its documented effect. Acetohexamide had minimal effect in the reversal of XP-C phenotype when tested in our screen, yet we managed to identify other drugs that can aid in this phenotypic partial reversal. Acetohexamide enabled the degradation of MUTYH, a protein involved in the removal of adenine residues mispaired with 8-oxo-guanine residues after oxidative stress [35]. The authors hypothesized that the degradation of such protein might be enabling spatial access of the lesions to other repair machinery independent of nucleotide excision repair [25] which was not yet further explored to date. It is therefore possible that the hit drugs identified in this study might similarly impose spatial access effect or that they interfere with one or several mediators of DNA repair, a hypothesis which needs to be further on examined.

Collectively, our data show that isoconazole and clemizole hydrochloride mediated an increase in cell viability post UV irradiation and enabled to a lower extent the repair of accumulated DNA damage in the form of UVB-induced 6-4PP. Isoconazole, on the one hand, mediated a curative effect post UV irradiation by enabling the decrease of apoptotic cells’ population as measured by flow cytometry with Cell Event caspase 3/7 activity staining. Clemizole hydrochloride’s effect, on the other hand, was both preventative pre UV and curative post UV irradiation showing a *p*-value < 0.05, while the difference between the post-irradiation alone and the pre-irradiation treatment was not significant. Such an effect is mediated via a yet unknown mechanism. The simultaneous double treatment with both drugs revealed no synergistic or additive effect. This suggests that both drugs interfere with a similar biological process or target which is consistent with the fact that they share a similar chemical scaffold (azole ring). Moreover, both drugs were shown not to affect the cells’ proliferation rate. The photoprotective effect of the drugs was also evident in WT cells post UV irradiation signifying the involvement of a protective pathway indirectly related to the mutated *XPC* gene. This work will mark the first attempt in discovering compounds for the amelioration of the XP-C phenotype, yet the exact regulated target needs to be further on explored to allow the identification of the effectors aiding in this phenotypic reversal and thus paving a way for a potential therapy for this yet untreatable genodermatosis.

## 4. Materials and Methods

### 4.1. Cell Line

Wild type (AG10076) and XP-C (GM15983) immortalized patient derived-fibroblasts were purchased from Coriell Biorepository. XP-C fibroblasts possess a two-base pair shift mutation at codon 431 of the *XPC* gene. The cells were cultured in DMEM high glucose, GlutaMAX media (Gibco, Waltham, MA, USA) supplemented with 10% FBS and 1% penicillin/streptomycin at 37 °C in a 5% CO_2_ incubator.

### 4.2. Chemical Drug Screening

The Prestwick chemical library was utilized. It consists of 1280 drugs of high chemical and pharmacological diversity. The drugs are all approved (FDA, EMA or other agencies). The drugs cover all main ATC groups and are dedicated to either CNS, cardiovascular, metabolism or infectiology diseases with either enzymatic or GPCRs targets. The library was screened at a final concentration of 10 µM. For the screening procedure, the cells were incubated with the drugs for 24 h then media was recuperated and the cells washed with PBS. Irradiation was carried out in the presence of PBS. Following that same drug, containing media were returned back to the cells for the course of post treatment to be incubated for further 24 h before the assessment of the readouts.

### 4.3. UV Dose-Response

To examine the photosensitivity of XP-C cells relative to WT in response to UVB irradiation, both cells were seeded in 96 well plates until 80% confluency, washed with PBS, then subjected to increasing doses of UVB. Twenty-four hours post UV, the viability of the cells was recorded as a measurement of the cells’ reducing capacity by PrestoBlue (Thermofisher Scientific, Waltham, MA, USA) according to the manufacturer’s suggestion. The LD50 for each cell line was calculated.

### 4.4. XPC Immuno-Staining and Associated Microscopy

For the characterization of XPC expression, both cell lines were fixed with 4% paraformaldehyde followed by permeabilization using 0.2% Triton X-100 and saturation with 3% FBS in PBS. Primary antibody targeting XPC (mouse monoclonal, Thermofisher Scientific, Waltham, MA, USA) was utilized followed by incubation with Alexa Fluor 488-coupled secondary antibody (goat anti-mouse, Invitrogen, Waltham, MA, USA). Nuclear DNA was counter-stained with Hoechst (Sigma-Aldrich, Burlington, MA, USA). Cell images were acquired by the Zen Axio-observer at 40X.

### 4.5. LC-MS/MS DNA Damage Quantification

The quantification of pyrimidine dimers (CPD & 6-4PP) was carried out by LC-MS/MS according to previous protocol [36]. Briefly, both cell lines were seeded in 100 mm dishes until sub-confluency where they were subjected to a UVB dose of 0.01 J/cm^2^. At different time points post UV (0, 2, 24 and 48 h), the cells were collected and their DNA extracted with Qiagen DNEasy Kit (Hilden, Germany). Sample preparation was carried out in two steps; the first includes incubation of DNA for 2 h at 37 °C and pH 6 in the presence of nuclease P1, DNase II and phosphodiesterase II. The pH was then raised to 8 by the addition of Tris. A second incubation period in the presence of phosphodiesterase I and alkaline phosphatase at 37 °C for 2 h yielded digested DNA with normal bases as nucleosides and photoproducts as dinucleoside monophosphates. Samples were then injected on an HPLC system (Agilent, Massy, France) connected to a reverse-phase HPLC column (150_2mm ID, 5 mm particle size, ODB, France). The detection was provided first by a UV detector aimed at quantifying normal nucleosides. Photoproducts were detected by a tandem mass spectrometer (API 3000, SCIEX, Thorn hill, ON, Canada) used in the reaction-monitoring mode as previously described [37]. The four CPDs and the four 6-4PPs (TT, TC, CT and CC derivatives) were quantified individually in the same HPLC run. Results were expressed in the number of lesions per million normal bases.

### 4.6. 6-4PP DNA Damage Staining and Quantification

For the quantification of DNA damage, cells were seeded until confluency, then subjected to UVB irradiation at a dose of 0.02 J/cm^2^. Twenty-four hours post UV, the cells were stained according to a previous protocol [38], consisting first of cell fixation with 4% paraformaldehyde, and permeabilized with 0.2% Triton X-100. Cells were further on treated with 2M HCL to denaturate the DNA and thus allow access to the DNA damage-targeting antibody. After saturation, the cells were incubated with 6-4PP antibody (64M-2 cosmo bio, Japan) followed by AF488 secondary antibody (Invitrogen, Waltham, MA, USA) and the DNA was counterstained with Hoechst (Sigma Aldrich, Burlington, MA, USA). Image acquisition and quantification were carried out by a Cell-insight NXT high content screening platform at 10X magnification. Single-cell fluorescence was quantified for all the cells under each particular treatment condition.

### 4.7. Ki67 Staining and Single-Cell Quantification

The cells were seeded until sub-confluency, then subjected to UVB irradiation at a dose of 0.02 J/cm^2^. Twenty-four hours post-irradiation, the cells were fixed with 4% paraformaldehyde and permeabilized with 0.2% Triton X-100. After saturation, the cells were incubated with ki67 antibody (clone MIB-1, Clinisciences, France) followed by AF488 secondary antibody (Invitrogen, Waltham, MA, USA), and the DNA was counterstained with Hoechst (Sigma Aldrich, Burlington, MA, USA). Image acquisition and quantification were carried out by Cell-insight NXT high content screening platform at 10X magnification. Single-cell fluorescence was quantified for all the cells under each particular treatment condition.

### 4.8. EdU Cell Proliferation Assay

Cell health and genotoxicity can be assessed by measuring the cell’s ability to proliferate via the incorporation of the nucleoside analog EdU. The latter can be further on quantified by a click-it covalent reaction between an azide and alkyne catalyzed by copper. For that, both cell lines were seeded in 100 mm dishes subjected to different forms of treatment followed by irradiation. Sham irradiated and sham-treated controls were also utilized. Two hours before the end of post UV incubation, EdU was diluted in the cell media at different time points. The cells were then collected and stained according to the manufacturer’s protocol. The samples were analyzed by flow cytometry (FACScan, BD LSRII flow cytometer, BD Biosciences, Franklin Lakes, NJ, USA). The post analysis was carried out using flowing software [39] (Turku Bioimaging, Finland).

### 4.9. Cell Event-PI Cell Death Quantification

To follow the effect of treatment on the induction of apoptosis post UV irradiation, CellEvent (Thermofisher Scientific) was utilized, which allows the measurement of caspase 3/7 activity. For that, cells were seeded in 100 mm dishes, treated with the molecules, irradiated, then Cell Event was added post UV and incubated for 24 h. The cells were then collected and PI was added before their analysis by flow cytometer (FACScan, BD LSRII flow cytometer, BD Biosciences). The post analysis was carried using flowing software [39] (Turku Bioimaging, Finland).

### 4.10. Statistical Analysis

Drug screening hit selection and single-cell analysis was carried out using R software [40]. Statistical analysis was conducted using GraphPad Prism v.8 after data normalization and quantification of normality to allow the choice of the respective statistical test (parametric or non-parametric) for each particular set of experiments.

## Data Availability

Not applicable.

## Supplementary Materials

The following are available online at https://www.mdpi.com/article/10.3390/ijms22158156/s1, Figure S1: Primary Screen UV dose optimization and reproducibility assessment. Figure S2: Single-cell analysis of nuclear DNA damage quantification. Figure S3: Drug treatment has no effect on ki67 proliferation marker expression. Figure S4: Isoconazole and not clemizole hydrochloride decreases the population % of apoptotic WT cells post UV.
