# Supplementary material for: Isoconazole and Clemizole Hydrochloride Partially Reverse the Xeroderma Pigmentosum C Phenotype
Source: Int J Mol Sci. 2021 Jul 29;22(15):8156. doi: 10.3390/ijms22158156 (PMC8346964; doi:10.3390/ijms22158156)
Supplement: Supplementary file 1 [file ijms-22-08156-s001.zip › ijms-1289503-supplementary.pdf]

Supplementary Material

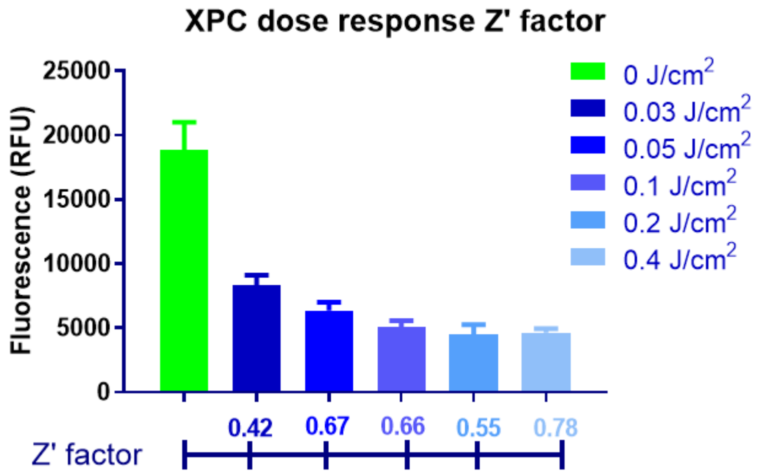

Figure S1. Primary Screen UV dose optimization and reproducibility assessment. To determine if the data collected from each plate meet the minimum quality requirements, quality control assessment was carried out with the controls. The latter include DMSO treated UV irradiated or protected cells. The calculation of the Z' factor was performed at the different doses to allow the selection of the appropriate dose giving a Z' factor above 0.5, without the induction of excessive cell death. This Z' factor signifies the applicability of the screen on large scale. Moreover, having a good separation between the positive and negative is a hallmark for cell reproducibility, for that several UV doses where tested. The dose 0.05j/cm^2 was chosen as it is the lowest lethal dose with a Z' factor above 0.5.

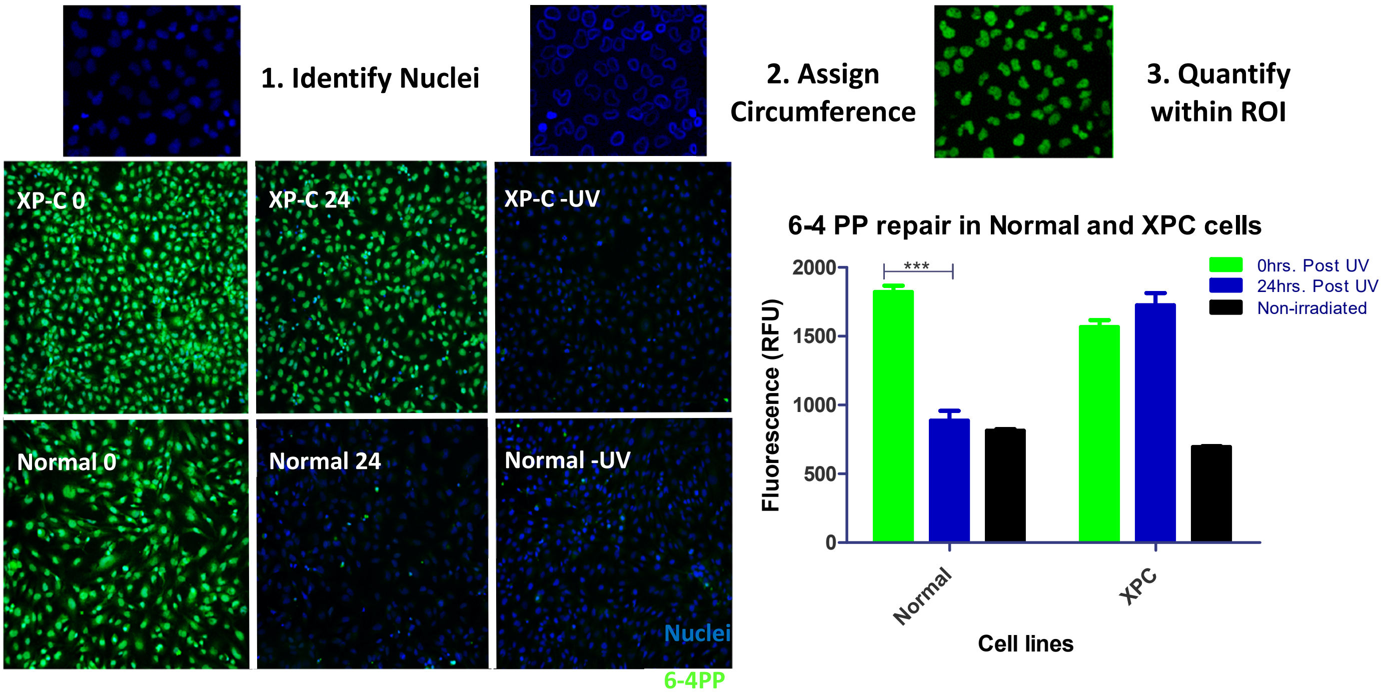

Figure S2. Single-cell analysis of nuclear DNA damage quantification. UVB induces the formation of two types of direct DNA damages that are the cyclobutane pyrimidine dimers (CPD) and the 6-4 pyrimidine pyrimidone photoproducts (6-4PP). CPD lesions require 48hrs to be repaired while 6-4PP are mostly removed 2hrs post UV irradiation in WT cells while they accumulate in XP-C cells without repair. The quantification of DNA damage post-treatment allows the identification of molecules that can decrease such damage in XP-C cells. 6-4 PP quantification was carried on non-treated irradiated or non-irradiated WT and XP-C cells. XP-C cells show elevated levels of DNA damage 24hrs. post UV while normal cells show significant complete repair manifested with minimal DNA damage lesions detected after the same time point. The analysis is based on single-cell quantification of the fluorescence signal for better statistical value. This is carried out by utilizing Hoechst staining for the identification of the nuclei that will be set as the region of interest (ROI) for the quantification of DNA damage within the circumference of the nucleus of each individual cell. *** $p<0.001$ paired t test.

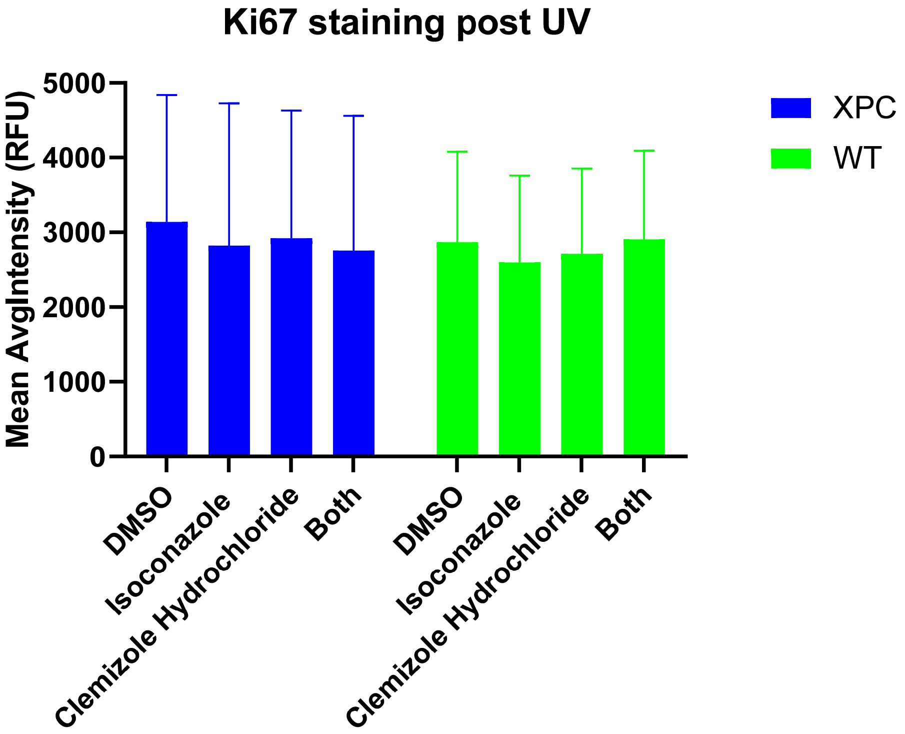

Figure S3. Drug treatment has no effect on ki67 proliferation marker expression. XP-C and WT cells were treated with isoconazole, clemizole hydrochloride, both or with DMSO then irradiated. 24 hours post UB the cells were fixed and stained with ki67 Ab. The staining profile of the proliferative marker ki67 was the same for all treatment conditions in both XP-C and WT cells.

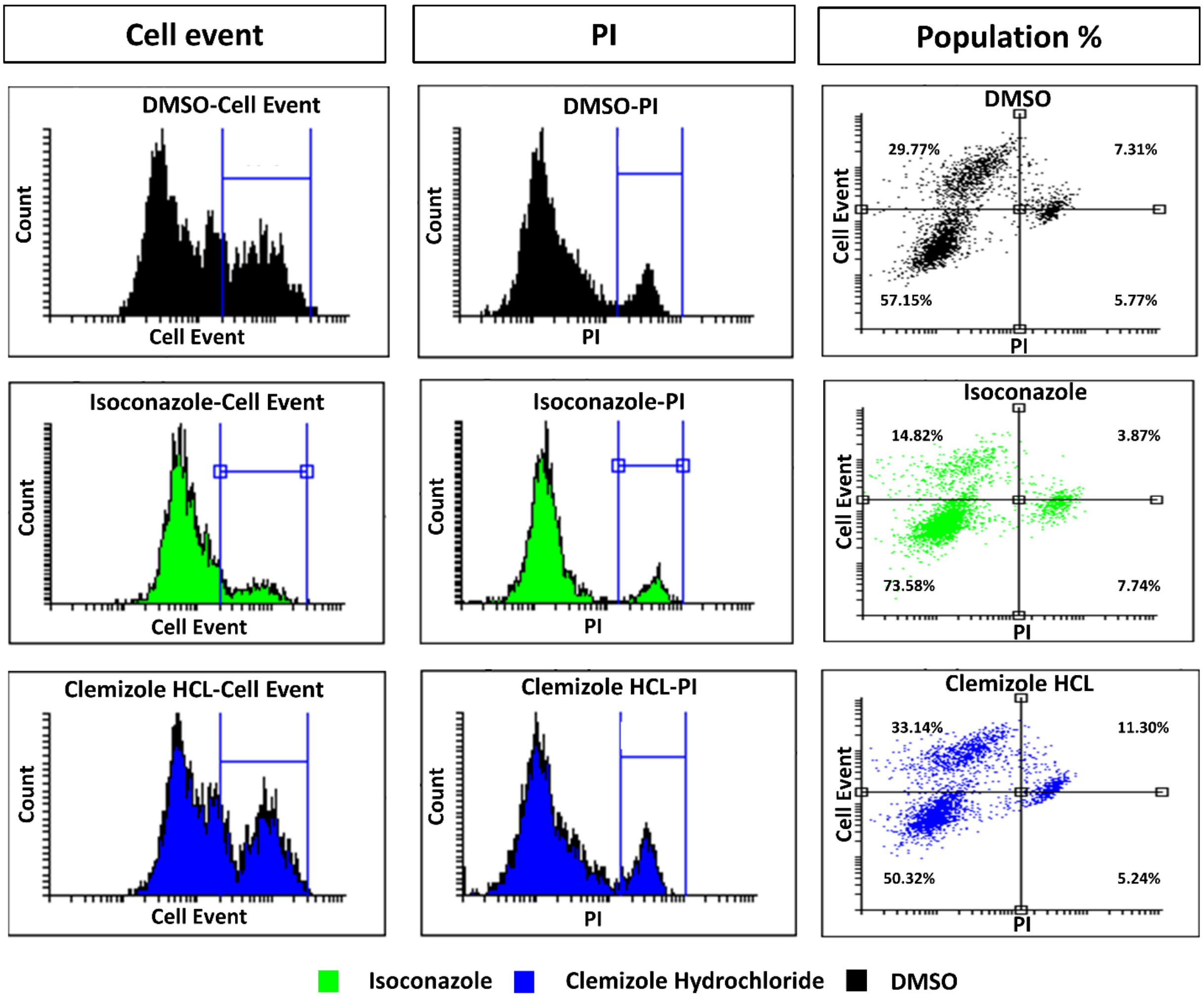

Figure S4. Isoconazole and not clemizole hydrochloride decreases the population % of apoptotic WT cells post UV. WT cells were treated with the different drugs or DMSO then irradiated. Cell event caspase 3/7 marker was added post UV then cells were collected. PI was added just before analysis on flow cytometer. Isoconzaole treatment decreased the percentage of apoptotic cells post UV (14.82%) compared to DMSO treated cells (29.77%). Clemizole hydrochloride, however, had no effect so it is perhaps via another mechanism that this drug mediates its mode of action.
